# Potential of Breast Milk Exosomes in Modulating Infant Developmental Programming: A Multi-Omics Study Based on a Birth Cohort

**DOI:** 10.3390/nu18132058

**Published:** 2026-06-24

**Authors:** Ying Lyu, Yalin Zhou, Xiaoyu Zhu, Muke Han, Wanyun Ye, Qiaosi Wei, Shilong Jiang, Kaifeng Li, Yajun Xu

**Affiliations:** 1 Department of Nutrition and Food Hygiene, School of Public Health, Peking University, Beijing 100083, China; lybjmu@126.com (Y.L.); zylyingyang@163.com (Y.Z.); 2211110219@stu.pku.edu.cn (X.Z.); hanmuke33@163.com (M.H.); yewanyun_vera@163.com (W.Y.); wqiaosi@163.com (Q.W.); 2 PKUHSC-China Feihe Joint Research Institute of Nutrition and Healthy Lifespan Development, Beijing 100083, China; jiangshilong@feihe.com; 3 Feihe Research Institute, Heilongjiang Feihe Dairy Co., Ltd., Beijing 100016, China

**Keywords:** human breast milk, exosome, multi-omics, adipogenesis, neurodevelopment

## Abstract

Background: Human breast milk (HBM), as the initial food for humans, is quite essential for infant development and also for health throughout the lifespan. Exosomes are bioactive components in HBM, yet their nutritional role remains poorly recognized. Objectives: This study investigates how HBM exosomes change with lactation and their potential role in infant growth and development. Methods: HBM samples were obtained at 2 and 6 months postpartum from a well-established birth cohort. Purified exosomes were detected using transcriptomic, lipidomic, and proteomic approaches. Then, multi-omics data were analyzed to compare differentially expressed miRNAs, lipids, and proteins along with different lactation periods and their association with the infant growth process. Results: Compared with the 2-month postpartum group, the expression levels of miR-214-3p, miR-199a-5p, miR-126-3p, miR-127-5p, miR-144-3p, and miR-4787-5p were down-regulated in the 6-month postpartum group. In addition, 190 lipids and 269 proteins were up-regulated in the 6-month postpartum group, whereas 15 lipids and 244 proteins were down-regulated. Enrichment analysis revealed that the predicted target genes of differentially expressed miRNAs were primarily involved in cell communication and axon guidance. In parallel, the differentially expressed proteins were enriched in biosynthesis of unsaturated fatty acids and fatty acid metabolism pathway, implying a potential role in adipogenesis and neurodevelopment. Conclusions: This study reveals that the cargo contents of HBM exosomes change with the lactation period and may adapt to the needs of infant growth and development, particularly adipogenesis and neurodevelopment. HBM exosomes may play an important role in transferring genetic information from mothers to infants and be related to infants’ development. The underlying mechanisms warrant further investigation and validation.

## 1. Introduction

Human breast milk (HBM) is the natural and ideal food for infants. The WHO and UNICEF recommend that infants should be exclusively breastfed for the first 6 months of life, as HBM supplies all the necessary energy and nutrients during this period [1]. However, even in well-nourished mothers, physiological and environmental factors may in some cases compromise nutrient status and the capacity for nutrient transfer via breast milk [2].

Breastfeeding provides immediate postpartum benefits for mothers, including reduced infectious symptoms, attenuated stress responses, lower blood pressure and weight [3]. In the long term, it reduces the mother’s susceptibility to breast and ovarian cancer, cardiovascular diseases and type 2 diabetes [4]. Beyond its physical advantages, breastfeeding enhances maternal psychological well-being, especially preventing postpartum depression [5]. HBM confers broader protection to infants compared with formula-feds, including improved growth patterns and nutritional status, reduced infection frequency, as well as favorable long-term outcomes in neurodevelopment and lower prevalence of obesity, diabetes, and cardiovascular disease [6].

HBM not only provides energy and nutrients including proteins, fats, carbohydrates, minerals, and vitamins for infant growth, but also contains bioactive components [7], such as oligosaccharides, immunoglobulins, leptin, lactoferrin, osteopontin, cytokines and so on [8]. Oligosaccharides may nourish probiotics in the gut and modulate cellular functions [9]. Cytokines may be involved in regulating immune maturation [10]. Secretory immunoglobulin A has been found to regulate both microbial colonization and infant immune responses [11]. Furthermore, HBM composition changes dynamically throughout the lactation period to meet the evolving needs of infant growth at different stages [12]. Colostrum is produced only for approximately 5 days postpartum. Although secreted in low volumes, it contains much higher levels of IgA, lactoferrin, cytokines, and growth factors compared with later stages, thereby exhibiting enhanced immune-protective and gut-modulating properties [7]. Transitional milk appears between 6 and 15 days postpartum. It is produced in larger amounts and has higher levels of lactose, fat, and water-soluble vitamins. HBM is considered almost mature from the 15th day of lactation and fully mature by 4 to 6 weeks postpartum [13].

Exosomes are regarded as a novel type of a bioactive component in HBM, as they have been demonstrated to exert regulatory functions through their cargoes [14]. Exosomes are extracellular nanovesicles with a diameter ranging from 40 to 160 nm. They are involved in a variety of biological processes, including immune modulation, stress adaptation, and inflammatory responses. Additionally, they serve as key mediators of intercellular signaling and communication [15]. HBM exosomes originate from the mammary gland epithelial cells [16], which are encapsulated in the phospholipid bilayer plasma membrane and contain a wide variety of cargoes including proteins, nucleic acids, lipids and metabolites [17]. It is now recognized that exosomes exhibit bioactivities through transporting those cargoes, among which miRNAs attract more attention for their epigenetic programming regulation function [18].

HBM is one of the richest sources of miRNAs in comparison with other human bodily fluids [19]. miRNAs are short, non-coding RNA sequences of approximately 22 nucleotides that act as post-transcriptional regulators of gene expression by degrading complementary mRNA in association with the Argonaute complex. Recent results suggest that both positive and negative regulation of transcription by miRNAs may occur in different tissues [20]. Thus, exosomal miRNAs in HBM carrying enormous genome information can be transferred from mother to infant via breastfeeding. The bilayer membrane of exosomes allows them to survive gastric digestion intact. Consequently, they are taken up by infant intestinal cells and transported via bloodstream to distant target tissues for biological action and infant gene expression regulation [6,21]. This may provide a clue to reveal how breastfeeding plays a role as maternal-to-child biochemical communication with intergeneration health consequences, while the mechanisms are not yet fully understood [22].

In contrast to miRNAs, studies analyzing lipids and proteins in HBM exosomes are far fewer, and no multi-omics analysis has been conducted. Martijn et al. [23] reported a total of 1963 proteins were identified in milk-derived extracellular vesicles purified from mature milk samples collected from a small sample size of seven healthy mothers. Ye et al. [24] detected a total of 11 lipid species and 182 molecular species across different lactation periods. In addition, whether HBM exosomal cargo undergoes dynamic changes across the lactation period to support infant growth and development has not been adequately investigated. Thus, we conducted an exploratory study using a birth cohort integrated with multi-omics analysis. This study intends to establish the first multi-omics profile of HBM exosomes, preliminarily characterize the properties and potential biological functions of HBM exosomes, and explore the potential associations between HBM exosomes and infant growth and development during the first 6 months postpartum.

## 2. Materials and Methods

### 2.1. Compliance with Ethics Requirements

The research was conducted according to the ethical policies and procedures approved by the Shanghai Nutrition Society Ethics Committee (Approval No.2016 (006), Date: 28 September 2016), and written informed consent was provided by all participants.

### 2.2. Study Population Recruitment and HBM Collection

The present study is based on the birth cohort of the Chinese Human Milk Project (CHMP), the study design of which has been described in a previous article [25]. Healthy lactating mothers aged 25 to 35 years who were breastfeeding infants between 15 and 180 days old, and who were non-smokers and non-drinkers were recruited. A total of 100 healthy lactating participants with term delivery and exclusive breastfeeding practices were randomly selected from the CHMP study population under matching conditions in demographic characteristics, economic status and dietary habits, with 50 collected breast milk samples at 2 months postpartum (PN-2M) and 50 at 6 months postpartum (PN-6M).

Breast milk samples were collected from each of these participants at 9:00–11:00 in the morning of the sampling day, at least one hour after the first feeding. The nipple and areola were cleansed. A single breast was completely emptied using a sterile electric pump. The breast milk was gently mixed to ensure homogenization and then carefully collected. A total of 30 mL were taken out and placed into a sterile test tube and the remaining samples were aliquoted to 10 mL tubes as needed. All aliquoted samples were transported to our laboratory on dry ice, then fast-frozen with liquid nitrogen and preserved at −80 °C for future exosome isolation and multi-omics test. Maternal and infant characteristics and health information were collected from the cohort sample database.

### 2.3. Isolation and Identification of Exosomes

The isolation of exosomes was referenced from a previous study [16]. The frozen HBM samples were thawed at 37 °C and subjected to serial centrifugations using refrigerated centrifuge (Microfuge 20R, Beckman, Brea, CA, USA) at 2000× *g* and 4 °C for 10 min to remove milk fat. The resultant supernatants were transferred to ultracentrifuge tubes and centrifuged at 10,000× *g* and 4 °C for 30 min to remove the remaining fat and cell debris. This procedure needed to be repeated twice. The supernatants were collected for another centrifugation with ultracentrifuge (CP100MX, Hitachi, Tokto, Japan) at 100,000× *g* and 4 °C for 70 min to yield a crude exosome pellet. The obtained pellet was suspended in 1 mL phosphate buffer saline (PBS) and stored at 4 °C. A total of 1 mL of the exosome sample was slowly layered on top of 250 μL of a 30% sucrose cushion (made with D_2_O) and ultracentrifuged at 100,000× *g* for 70 min. A total of 250 μL of the lower 30% sucrose cushion was retrieved and diluted to 3 mL with PBS, followed by centrifugation at 100,000× *g* for 70 min. Finally, the supernatant was removed. The obtained pellet of purer exosome precipitates was resuspended in 200 μL of ice-cold PBS.

Identification of exosomes was confirmed by the transmission electron microscope (TEM) (HT-7700, Hitachi, Tokyo, Japan) and high-sensitivity flow cytometry. Briefly, the samples containing resuspended exosomes in PBS were stained for 1 min with 1% uranyl acetate. Excess fluid was removed with a piece of filter paper. All transmission electron micrographs were obtained using electron microscopy at 100 kv. High-sensitivity flow cytometry for nanoparticle analysis was used to determine the size distribution and concentration of exosomes and fluorescent labeling of exosomes. Take 30 μL of the diluted exosomes and mix it with 20 μL of fluorescently labeled antibody CD9, CD63 and CD8, respectively. Incubate the mixtures at 37 °C for 30 min in the dark. Add 1 mL of ice-cold PBS to each tube. Using an ultracentrifuge rotor, centrifuge the samples at 110,000× *g* and at 4 °C for 70 min. Remove the supernatants and repeat the centrifugation process once more. Carefully remove the supernatants and resuspend the pellet in 50 μL of ice-cold PBS. Prior to sample analysis, perform an instrument performance test using standard beads to ensure proper calibration.

### 2.4. Identification of miRNAs

miRNAs were identified by following the methods from Luan et al. [26]. Total RNA was extracted from HBM samples using Trizol Reagent (Invitrogen Life Technologies, Carlsbad, CA, USA) and concentration, quality and integrity were determined with a NanoDrop spectrophotometer (Thermo Scientific, Waltham, MA, USA). Small RNA sequencing libraries were prepared with the NEBNext Multiplex Small RNA Library Prep Set for Illumina (New England Biolabs Inc., Ipswich, MA, USA). In brief, 1 μg of total RNA from HBM samples was sequentially ligated to 3′ and 5′ adapters using a ligation enzyme mix. The mixture products were then reverse-transcribed with SuperScript II reverse transcriptase, followed by PCR amplification. A quality control (QC) test indicated the average size of inserts was approximately 140 to 150 bp. The sequencing library was quantified using the Agilent high-sensitivity DNA assay on a Bioanalyzer 2100 system (Agilent Technologies Inc., Palo Alto, CA, USA) and was then sequenced on the NovaSeq 6000 platform (Illumina, San Diego, CA, USA).

### 2.5. Lipid Extraction and Preparation for Lipidome

The lipid extraction procedure was carried out as described in a previous study [27]. Each sample was taken a portion and then added water to make it 200 μL, followed by vortexing for 60 s and sonicating for 2 min in ice water. Subsequently, 480 μL of extract solution (MTBE:MeOH = 5:1) containing internal standard was added. Samples were vortexed for another 60 s and sonicated for 10 min in ice water. After centrifugation at 3000 rpm for 15 min at 4 °C, 250 μL of supernatant was collected. A total of 250 μL of MTBE was added to the remaining sample, the vortex, sonication and centrifugation steps were repeated, and an additional 250 μL of supernatant was collected. This step was performed once more. The combined supernatants were dried in a vacuum concentrator at 37 °C. Then, the dried samples were reconstituted in 50 μL of resuspension buffer (DCM:MeOH:H_2_O = 60:30:4.5), the samples were vortexed for 30 s and sonicated for 10 min in ice water. The suspension was centrifuged at 12,000 rpm for 15 min at 4 °C, and 30 μL of supernatant was transferred to a fresh glass vial for LC/MS analysis. The QC sample was prepared by mixing equal volumes of supernatants from all individual samples.

### 2.6. LC-MS/MS Analyses of Lipidome

Lipid separation was performed on a SCIEX ExionLC series UHPLC System, as previously described [28]. Mobile phase A consisted of 40% water, 60% acetonitrile, and 10 mmol/L ammonium formate, while mobile phase B consisted of 10% acetonitrile, 90% isopropanol, and 10 mmol/L ammonium formate. The column temperature was maintained at 45 °C. A total of 3 μL of each sample was injected from an auto-sampler under 6 °C. AB Sciex QTrap 6500+ mass spectrometer was applied for detection with ion source parameters as follows: IonSpray voltage: +5500/−4500 V, curtain gas: 40 psi, temperature: 350 °C, ion source gas 1: 50 psi, ion source gas 2: 50 psi, decluster potential: ±80 V.

### 2.7. Protein Extraction and Preparation for Proteome

Protein preparation was carried out as described in the previous study [29]. Samples were lysed with 150 μL lysis solution and were ultrasonicated in ice water for 5 min. Following centrifugation at 12,000× *g* for 10 min at 4 °C, the supernatant was collected. A total of 10 μL of each sample were mixed together to build a sample library.

Protein concentration was determined using a bicinchoninic acid assay. Five times the volume of acetone was added to each sample of 20 μg total protein and precipitated overnight at −20 °C. The pellet was collected by centrifugation at 12,000× *g* for 10 min at 4 °C, then dissolved in 50 µL of reconstitution buffer and ultrasonicated in water for 3 min. Cooled the samples to room temperature. Proteins were reduced with dithiothreitol of 5 mM final concentration at 55 °C for 20 min. Then, the samples were incubated in the dark at room temperature for 30 min with iodoacetamide of 15 mM final concentration. Trypsin was dissolved with resuspension buffer to 0.5 μg/μL and added at a ratio of 1:50 (trypsin:protein, *m*/*m*) for overnight enzymolysis with shaking. The peptides were recovered with 2% trifluoroacetic acid by centrifugation at 12,000× *g* at room temperature for 10 min, 3 times. All the supernatant components were combined and centrifugated for 10 min, then the supernatant was transferred to a new tube to acquire peptide samples. Peptides were then desalted with buffer solution in C18 column and dried by vacuum overnight at 4 °C.

Testing samples and QC sample were dissolved using an iRT kit. A dried library-building sample was redissolved to 50 μL and fractionated into 12 fractions via high-pH reverse high-performance liquid chromatography. Then, the sample was dried by vacuum and the iRT kit was added.

### 2.8. Nano-LC-MS/MS Analyses of Proteome

Each sample of 2 μg total peptides was loaded onto an EASY-nLC 1200 nano-UPLC system. Separation was performed using mobile phase A of 0.1% formic acid in water-acetonitrile (98:2, *v*/*v*) and mobile phase B of 0.1% formic acid in acetonitrile-water (80:20, *v*/*v*). The eluted peptides were analyzed using Thermo Q-Exactive HFX equipped with a nano-electrospray ion source [29].

The parameter settings are referenced from a previous study [30]. For the library-building sample, data-dependent acquisition mode was used. The total analysis time was 90 min, adopting the positive ion detection mode. The full MS scans were acquired at a resolution of 120,000 for a scan range of 350–1600 *m*/*z*. Automatic gain control (AGC) was set as 3.0 × 10^6^ and with a maximum injection time (max IT) of 50 ms. High-energy collisional dissociation (HCD) fragmentation was performed at a normalized collision energy (NCE) of 27% and the isolation window was 1.6 *m*/*z.* MS/MS scans were performed at a resolution of 15,000 at 110 *m*/*z* with an AGC of 1.0 × 10^5^ and a max IT of 96 ms. For proteomics data collection of individual samples, data-independent acquisition was used. The total analysis time was 90 min, adopting the positive ion detection mode. The full MS scans were acquired at a resolution of 120,000 for a scan range of 350–1250 *m*/*z*. AGC was set as 3.0 × 10^6^ and with a max IT of 50 ms. HCD fragmentation was performed at a NCE of 27%. MS/MS scans were performed at a resolution of 15,000 at 110 *m*/*z* with an AGC of 1.0 × 10^6^ and a max IT of auto.

### 2.9. Analyses of Functional Enrichment

Gene ontology (GO) and Kyoto Encyclopedia of Genes and Genomes (KEGG) pathway enrichment analysis were performed on predicted target genes of differentially expressed miRNAs. GO enrichment was conducted using topGO (v2.50.0) based on a hypergeometric test (significant threshold was *p* < 0.05). KEGG pathway enrichment was performed using clusterProfiler (v4.6.0), with pathways meeting at *p* < 0.05 considered significantly enriched [31].

For GO functional enrichment, there are 3 functional categories: biological process, cellular component and molecular function, and the top 10 associated pathways in each category were exhibited according to the gene ratio. For KEGG functional enrichment, the result was displayed as the top 20 associated pathways according to the gene ratio.

### 2.10. Statistics

Statistical analysis was performed using the SPSS version 27.0. *p* values were calculated using the 2-sided *t*-test for continuous data and the Fisher exact test was used for categorical data. *p* < 0.05 was considered statistically significant.

DESeq (v1.39.0) was conducted to analyze the differentially expressed miRNAs; transcripts with |log2 fold change| > 1 and *p* < 0.05 were considered as differentially expressed miRNAs.

Biobud-v2.0.7 software was employed for the quantification of the target compounds. The absolute content of individual lipids corresponding to the internal standard was calculated based on peaks area and actual concentration of the identical lipid class IS. For the lipidomic data analysis, the SIMCA 16.0.2 software package (Sartorius Stedim Data Analytics AB, Umea, Sweden) was used for multivariate analysis. The data were scaled and logarithmically transformed to minimize the impact of both noise and high variance of the variables. After the transformations, principal component analysis (PCA) was performed to visualize the overall distribution and grouping of the samples. A 95% confidence interval in the PCA score plot was used as the threshold to identify potential outliers in the dataset. To visualize group separation and find significantly changed lipids between the two groups, supervised orthogonal projections to latent structures–discriminate analysis (OPLS-DA) was conducted. OPLS-DA summarized the contribution of each variable to the model. Furthermore, those with a variable importance in the projection value > 1 and *p* < 0.05 (Student’s *t*-test) were considered significantly changed lipids [32].

For the proteomic data analysis, the SIMCA 16.0.2 software package (Sartorius Stedim Data Analytics AB, Umea, Sweden) was used for multivariate analysis. The data were log-transformed and centered before the modeling analysis. Principal component analysis (PCA) was performed to visualize the overall distribution and grouping of the samples. The 95% confidence interval in the PCA score plot was used as the threshold to identify potential outliers in the dataset [30]. Differentially expressed proteins were selected by Student’s *t*-test or chi-square test. Proteins with *p* < 0.05 and |fold change| ≥ 2 were considered differentially expressed. Protein–protein interaction diagram (PPI) network analysis used Homo sapiens (human)-related data in the STRING database (v11, string-db.org).

Correlation network visualization was performed using the built-in Network Plot module of Origin Pro 2026 software (OriginLab Corporation, Northampton, MA, USA). Spearman’s rank correlation analysis was conducted on the original data to compute the correlation coefficient matrix, which served as the adjacency matrix for network construction. To retain meaningful and statistically significant associations, only correlations with absolute coefficient values |r| > 0.4 and *p* < 0.05 were preserved.

### 2.11. Chemicals

PBS (Sangon Biotech, Shanghai, China); FITC Mouse Anti-Human CD9 (BD, San Diego, CA, USA); FITC Mouse Anti-Human CD63 (BD, San Diego, CA, USA); FITC Mouse Anti-Human CD81 (BD, San Diego, CA, USA); Heavy water (Sigma-Aldrich, St. Louis, MO, USA); Trizol reagent (Invitrogen Life Technologies, Carlsbad, CA, USA); LC- and MS-grade methanol (CNW Technologies, Dusseldorf, Germany); LC- and MS-grade methyl tert-butyl ether (CNW Technologies, Dusseldorf, Germany); LC- and MS-grade ammonium acetate (Merck, Darmstadt, Germany); LC- and MS-grade acetonitrile (CNW Technologies, Dusseldorf, Germany); LC- and MS-grade dichloromethane (Merck, Berlin, Germany); LC- and MS-grade isopropanol (Merck, Berlin, Germany); LC- and MS-grade H_2_O (ANPEL Laboratory Technologies, Shanghai, China); SPLASH LIPIDOMIX mass spec standard (Avanti Polar Lipids, Alabaster, AL, USA); Dithiothreitol (Sigma-Aldrich, St. Louis, MO, USA); Iodoacetamide (Sigma-Aldrich, St. Louis, MO, USA); HEPES (2-[4-(2-hydroxyethyl)piperazin-1-yl] ethanesulfonic acid) (Sigma-Aldrich, St. Louis, MO, USA); Sodium deoxycholate (Sigma-Aldrich, St. Louis, MO, USA); Ammonium bicarbonate (Sigma-Aldrich, St. Louis, MO, USA); Anhydrous acetonitrile (Sigma-Aldrich, St. Louis, MO, USA); LC- and MS-grade formic acid (Sigma-Aldrich, St. Louis, MO, USA); Trifluoroacetic Acid (Sigma-Aldrich, St. Louis, MO, USA); Sequence grade trypsin (Promega, Madison, WI, USA).

## 3. Results

### 3.1. Characteristics of Mothers and Infants

Demographic and clinical characteristics of the 100 mothers and their infants who participated in this study are presented in Table 1. For mothers, we compared delivery age, pre-pregnancy BMI, gestational weight gain, the way of delivery, smoking status, and alcohol intake between group PN-2M and group PN-6M. There were no statistical differences observed among characteristics mentioned above. Over half of the mothers (60.00% in group PN-2M and 58.00% in group PN-6M) delivered their infants naturally per WHO recommendations. For their infants, differences in sex, birth weight and birth length between group PN-2M and group PN-6M did not reach statistical significance as well. Body weight, body length and head circumference at sampling in group PN-6M was significantly larger than that in group PN-2M along with infant growth, which reflects the normal physiological process of brain development in the early stages of human life.

### 3.2. Characterization of HBM Exosomes in Different Lactation Periods

**Figure 1:**
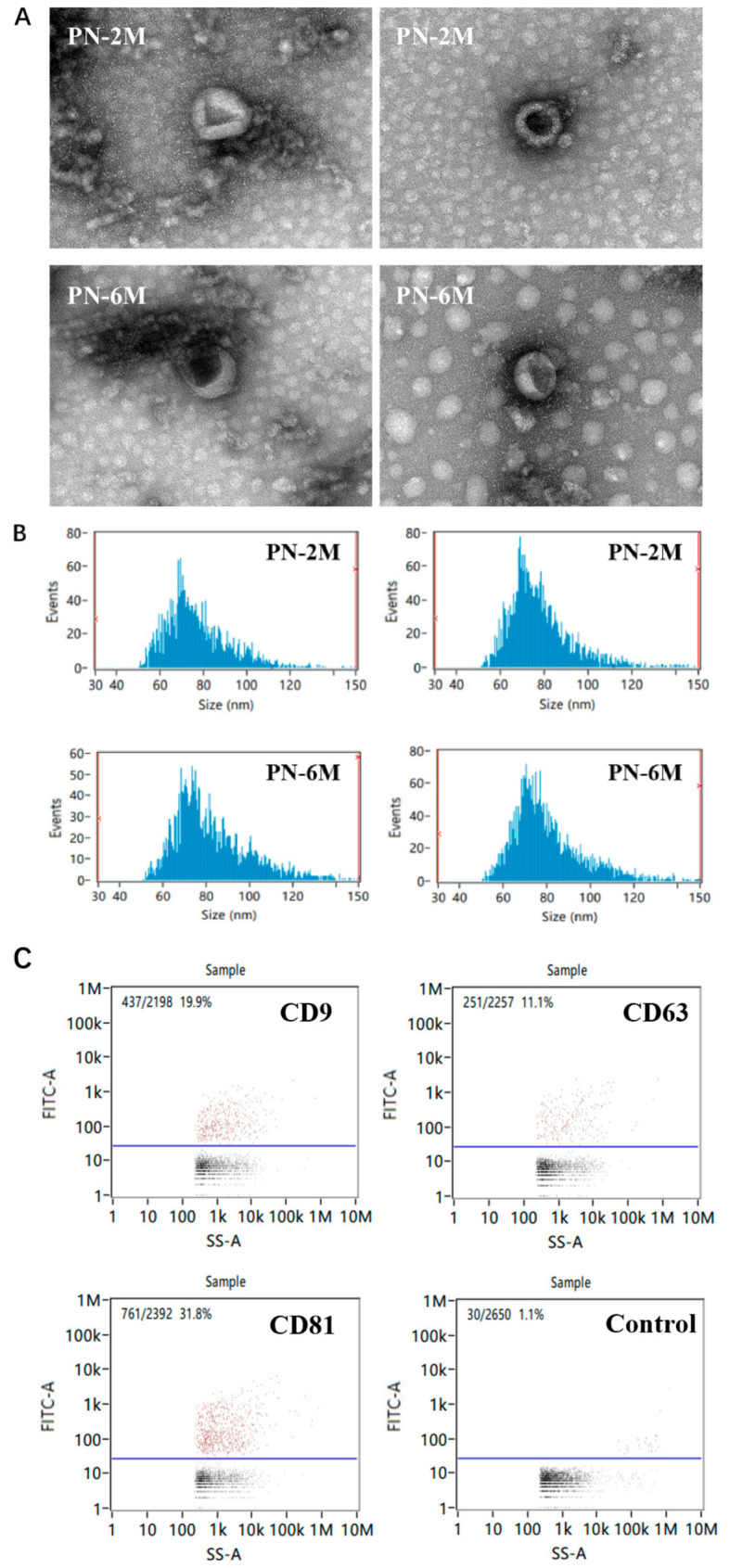
Characterization of HBM exosomes. (**A**) Transmission electron microscope image of HBM exosomes with 100 nm scale bar. (**B**) Particle size distribution of HBM exosomes using nanoparticle analysis. (**C**) The positive rate of exosome membrane surface protein expression of CD9, CD63, CD81 in HBM exosomes by fluorescence labeling and nanoflow cytometry.

The vesicular structure of isolated exosomes from HBM is shown under a 100 nm scale in Figure 1A. Different sizes of exosome-like vesicles were detected by the TEM. We examined all samples with high sensitivity flow cytometry for nanoparticle analysis. The diameter of HBM exosomes ranged from 50 to 150 nm (Figure 1B), with a median size of 73.36 nm, and an average of 77.14 ± 15.55 nm (means ± SD). The average concentration was 3.80 × 10^10^ particles/mL. We examined the exosome-specific markers of CD9, CD63, and CD81 of randomly selected 20 samples with fluorescence labeling and nanoflow cytometry (Table 2). All tested samples of two groups expressed membrane surface protein CD9, CD63, CD81 and had significant differences to control samples (Figure 1C).

### 3.3. miRNA Expression Analysis of HBM Exosomes

#### 3.3.1. miRNA Profiling of HBM Exosomes

**Figure 2:**
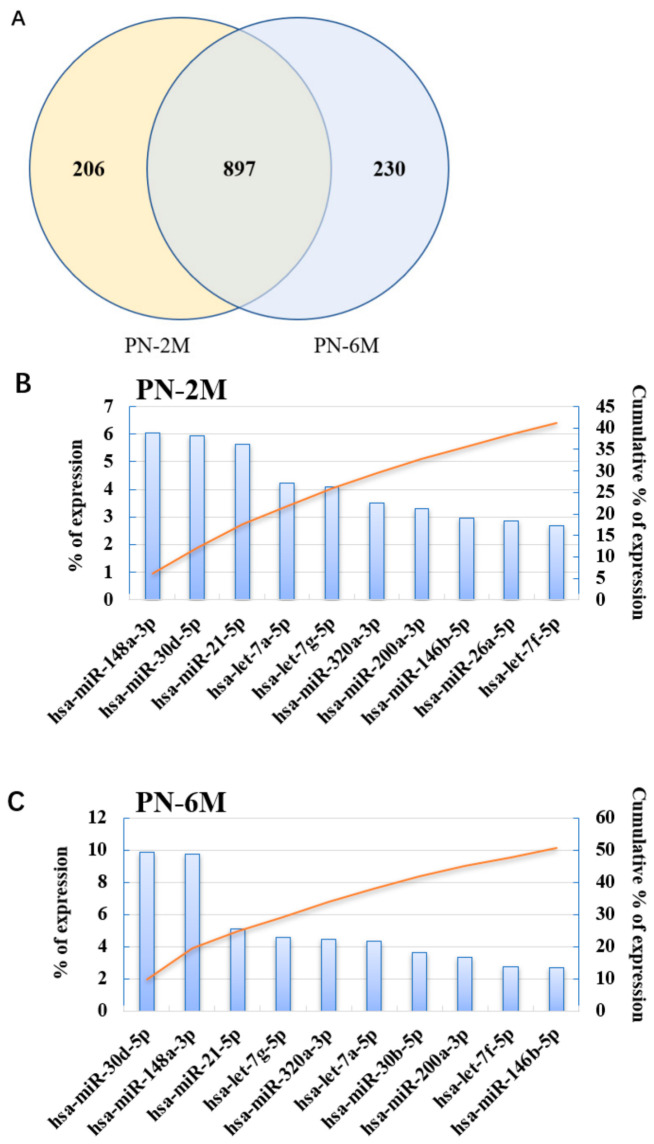
Expression and distribution of known miRNAs in HBM exosomes. (**A**) Venn diagram of miRNAs identified in group PN-2M and PN-6M. (**B**) Cumulative percentage of top 10 miRNAs expressed in group PN-2M. (**C**) Cumulative percentage of top 10 miRNAs expressed in group PN-6M.

In total, 1333 miRNAs were identified in HBM, among which 897 miRNAs exist in both group PN-2M and group PN-6M. A total of 206 miRNAs were only detected in group PN-2M and 230 miRNAs were only detected in group PN-6M (Figure 2A). The 10 most highly expressed miRNAs accounted for 41.2% and 50.5% in group PN-2M and PN-6M (Figure 2B,C). The top 10 abundant miRNAs in the two groups were almost the same with a different rank, except miR-26a-5p in group PN-2M and miR-30b-5p in group PN-6M. miR-148a-3p, miR-30d-5p, and miR-21-5p were the most highly expressed miRNAs in both groups.

#### 3.3.2. Differential miRNA Expression Analysis of Different Lactation Periods

**Figure 3:**
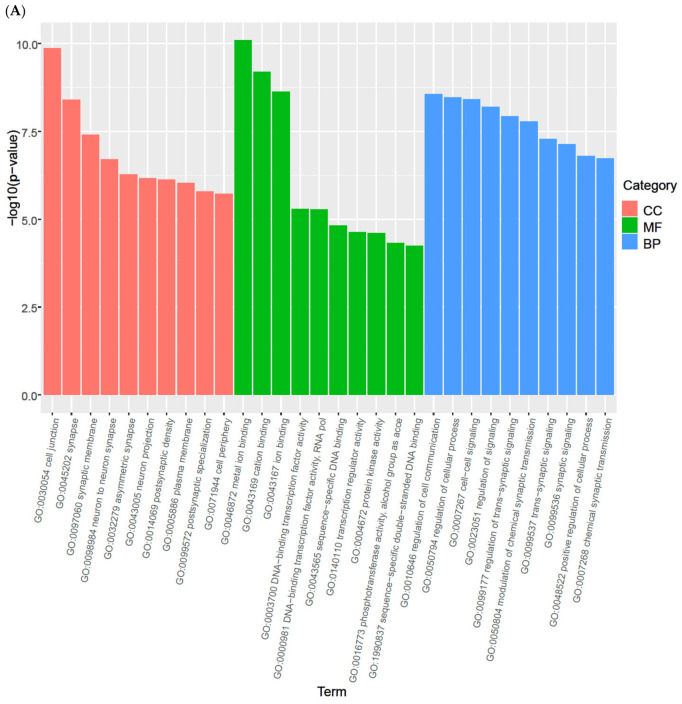

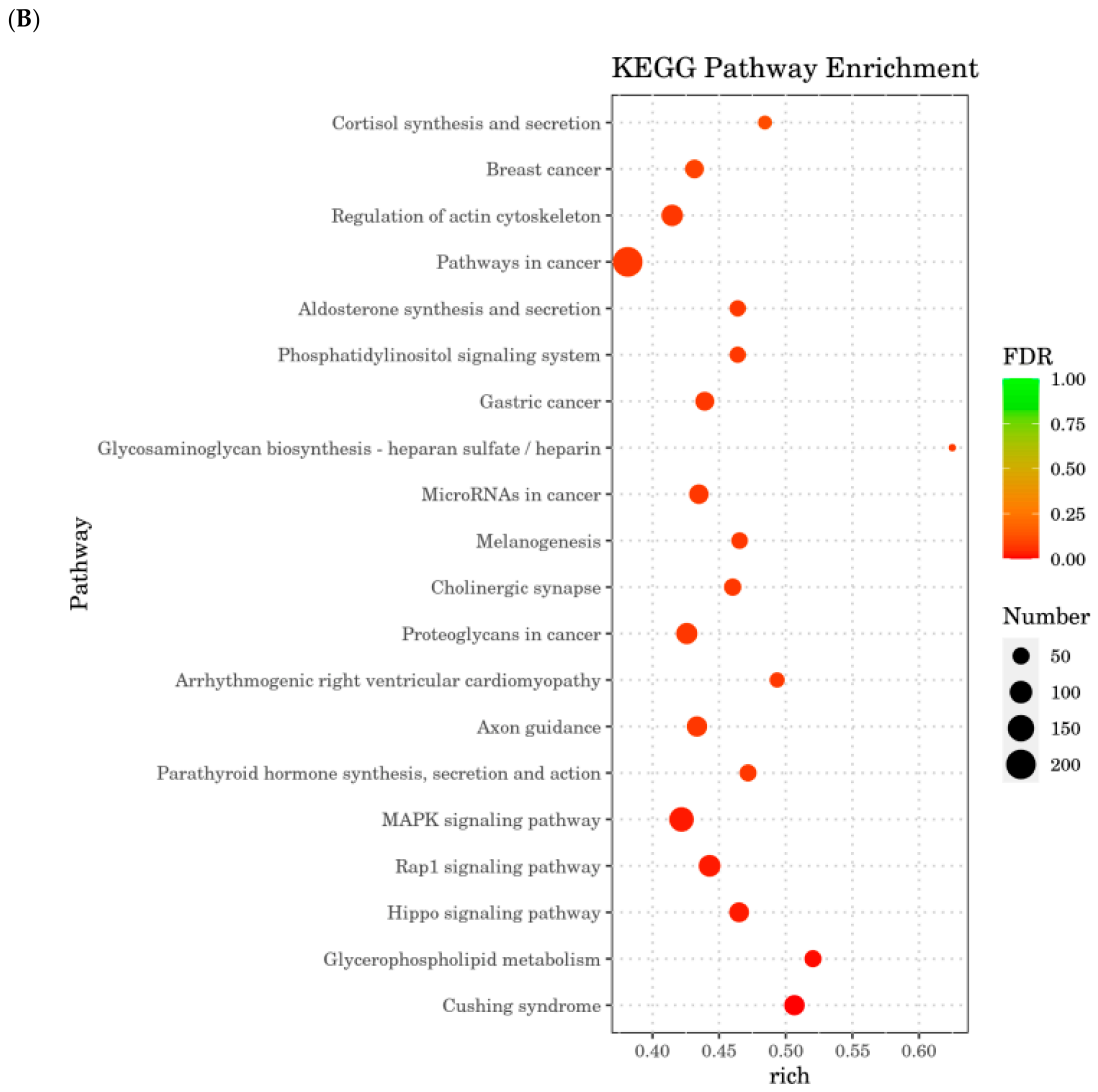
Enrichment analysis of differentially expressed miRNAs. (**A**) GO enrichment analysis of differentially expressed miRNAs. (**B**) KEGG pathway enrichment analysis of differentially expressed miRNAs.

The base mean value of differentially expressed miRNAs and fold changes are illustrated in Table 3. For all the miRNAs identified, six miRNAs were down-regulated in group PN-6M compared with PN-2M. They were miR-214-3p, miR-199a-5p, miR-126-3p, miR-127-5p, miR-144-3p and miR-4787-5p. The predicted target genes of differentially expressed miRNAs were subsequently included in GO classification and KEGG pathway analysis. As shown in Figure 3A, GO terms related to cellular components included cell junction, synapse, synaptic membrane, and neuron-to-neuron synapse. The top enriched molecular function included metal ion binding, cation binding and ion binding. GO terms related to biological processes focused on regulation of the cellular process, cell–cell signaling, regulation of signaling and several synaptic signaling-related processes. In addition, KEGG pathway enrichment analysis identified Cushing syndrome, glycerophospholipid metabolism, hippo signaling pathway, Rap1 signaling pathway and the MAPK signaling pathway (false discovery rate < 0.05), as Figure 3B demonstrates, which enriched 78, 51, 73, 93 and 124 candidate target genes respectively.

### 3.4. Lipidomic Analysis of HBM Exosomes

#### 3.4.1. Lipid Composition of HBM Exosomes

**Figure 4:**
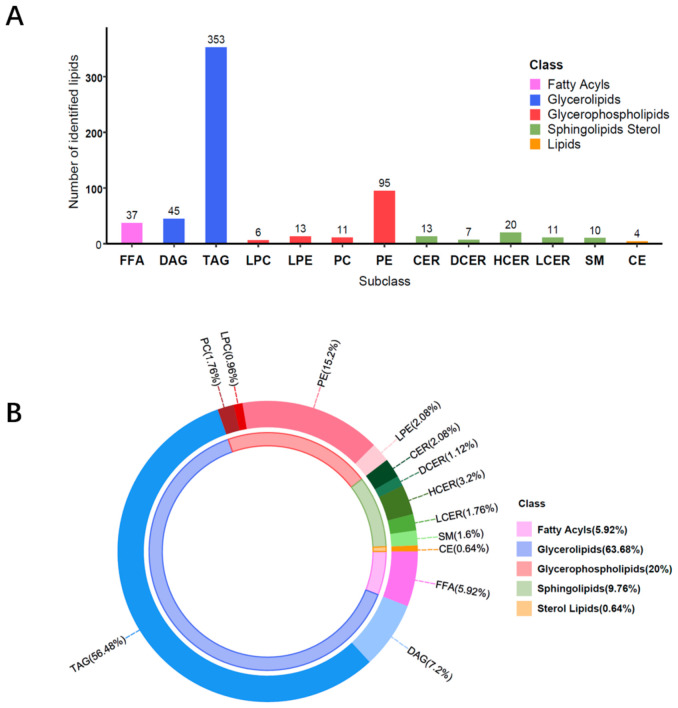
Classification of identified lipids in HBM exosomes. (**A**) Bar plot of number of identified lipids in subclasses. (**B**) Composition and percentage of each subclasses for total lipids. FFA, free fatty acid; DAG, diacylglycerol; TAG, tiacylglycerol; LPC, lysophosphatidylcholine; LPE, lysophosphatidylethanolamine; PC, phosphatidylcholine; PE, phosphatidylethanolamine; CER, ceramide; DCER, dihydroceramide; HCER, hexosylceramide; LCER, lactosylceramide; SM, sphingomyelin; CE, cholesteryl ester.

Lipids are classified into eight major categories based on their chemical composition [33]. In our study, we found five out of eight categories of lipids were detected in HBM exosomes, including fatty acyls, glycerolipids, glycerophospholipids, sphingolipids and sterol lipids. Prenol lipids, saccharolipids and polyketides were not detectable. The same 625 lipids sorted in 13 subclasses were identified in both group PN-2M and PN-6M (Figure 4A). Triglycerides (TAG) were the most abundant subclass of 353 lipids and phosphatidylethanolamines (PE) followed, with 95 lipids in total, accounting for 56.48% and 15.2% of the total 625 lipids, respectively (Figure 4B). To further characterize the TAG subclasses, the four most abundant molecular species were ranked identically in both groups: TAG(52:0)_FA18:0, TAG(50:0)_FA18:0, TAG(52:1)_FA18:1, and TAG(52:1)_FA18:0, listed in descending order of abundance.

#### 3.4.2. Exosomal Lipids Composition in Different Lactation Periods

**Figure 5:**
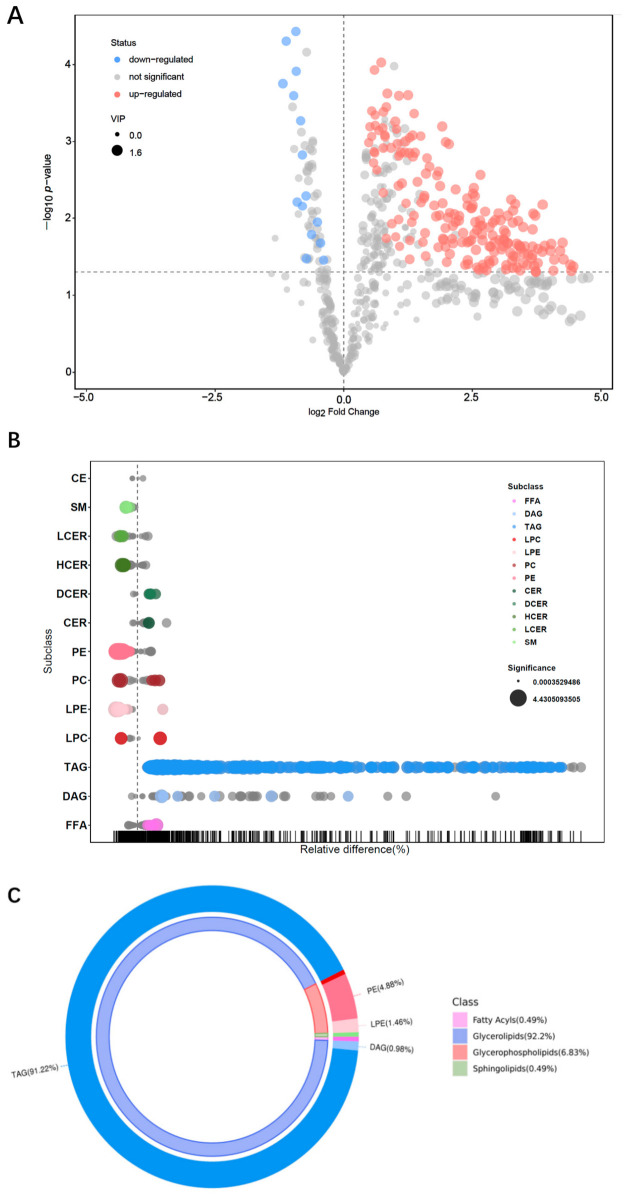
Comparison of lipids composition between group PN-2M and PN-6M. (**A**) Volcano plot analysis of identified lipids with blue dots representing down-regulated lipids and red dots representing up-regulated lipids in group PN-6M. (**B**) Bubble plot illustrates the relative difference in identified lipids. (**C**) Composition and percentage of each subclass for differential lipids.

Compared with group PN-2M, the 205 lipids that significantly varied in group PN-6M consisted of 190 up-regulated lipids and 15 down-regulated lipids (Figure 5A). Differential lipids were found in 12 subclasses, except in the CE subclass (Figure 5B). TAG was the most significantly varied subclass. A total of 91.22% of differential lipids belonged to the TAG subclass and all up-regulated were in group PN-6M. A total of 4.88% of differential lipids belonged to the PE subclass and were all down-regulated in group PN-6M (Figure 5C). The top 10 abundant lipids in the two groups are listed in Table 4. SM, especially SM(22:0), is the most abundant lipid in group PN-2M, while FFA(16:0) is the most abundant lipid in group PN-6M.

### 3.5. Proteomic Analysis of HBM Exosomes

**Figure 6:**
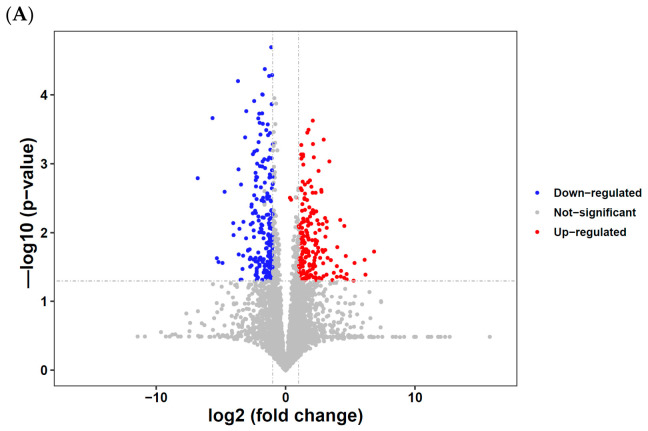

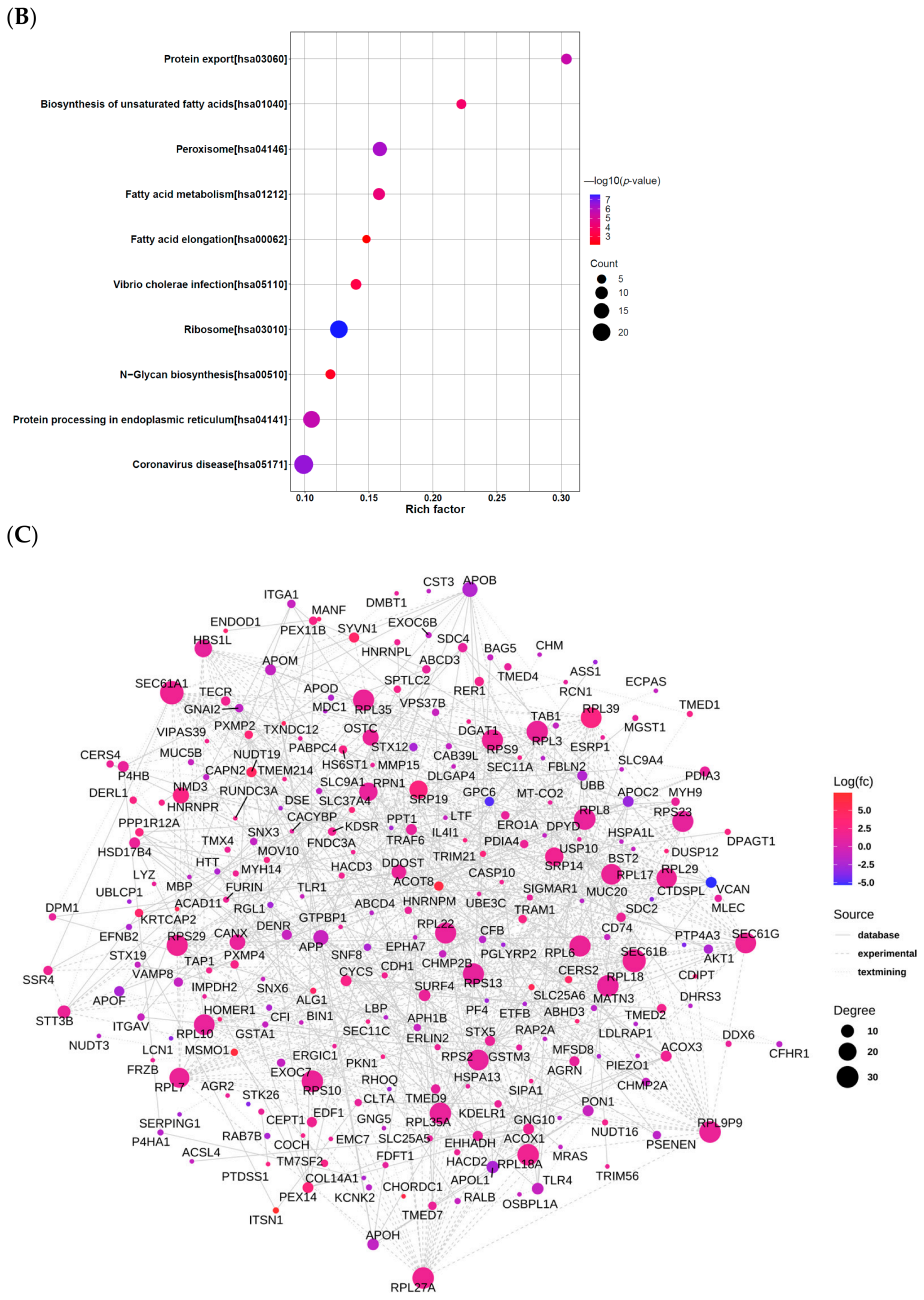
Proteomic analysis of differentially expressed proteins between group PN-2M and PN-6M. (**A**) Volcano plot of differentially expressed proteins in group PN-6M compared with PN-2M; (**B**) KEGG pathway enrichment analysis of differentially expressed proteins. (**C**) Differential expression PPI diagram.

A total of 4195 species of proteins were identified in the samples. Among them, 269 up-regulated proteins and 244 down-regulated proteins were discovered in group PN-6M compared with group PN-2M (Figure 6A). KEGG pathway enrichment analysis revealed that differentially expressed proteins are mainly associated with protein export, biosynthesis of unsaturated fatty acids, peroxisome, fatty acid metabolism and fatty acid elongation processes (Figure 6B). PPI showed that the genes/proteins interacting with SEC61A1 were the most numerous, reaching 35 (Figure 6C).

### 3.6. Multi-Omics Correlation Network Analysis

**Figure 7:**
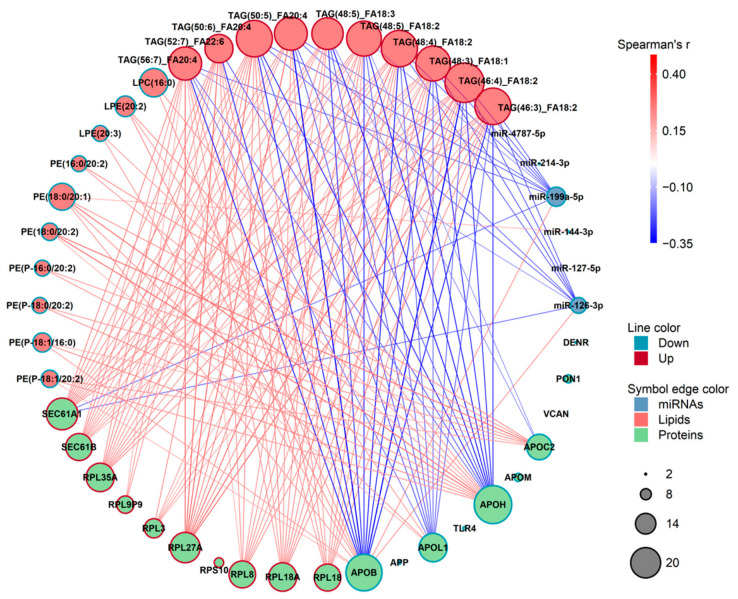
Correlation network analysis among differentially expressed miRNAs, lipids and proteins. Nodes in the plot represent each selected variable, and different node colors represent different categories (blue for miRNAs, red for lipids and green for proteins); the size of a node, defined as node degree, indicates the total number of nodes with significant associations with that node, and the higher the degree, the larger the node size; the color of the node edge represents the relative abundance change trend of the corresponding variable, where red indicates an increase in relative abundance and blue indicates a decrease in relative abundance. Edges represent significant correlations between variables that satisfy |r| > 0.4 and *p* < 0.05; the thickness of the edges is proportional to the absolute value of the correlation coefficient, and the higher the correlation strength, the thicker the edges; the color of the edges is used to distinguish correlation directions—red for positive correlation and blue for negative correlation.

Correlation network analysis and visualization were performed to comprehensively analyze the multi-omics data. For differentially expressed miRNAs, all six significantly down-regulated miRNAs were selected. For differentially expressed lipids, the 10 most up-regulated and 10 most down-regulated lipids were selected. For the differentially expressed proteins, selection was performed according to the number of connections (i.e., the degree of connectivity) in the PPI diagram. Specifically, among the up-regulated proteins, the 10 with the highest degree of connectivity were selected, and among the down-regulated proteins, the 10 with the highest degree of connectivity were also selected. The correlation network of the selected differentially expressed miRNAs, lipids, and proteins is presented in Figure 7. We found that most of the selected differentially expressed lipids and proteins were intercorrelated. Among the six differentially expressed miRNAs, miR-199a-5p, miR-126-3p and miR-144-3p were found to be associated with other selected differentially expressed lipids and proteins. Notably, both miR-199a-5p and miR-126-3p correlated with the proteins SEC61A1 and APOB, as well as most up-regulated TAGs of various chain lengths.

## 4. Discussion

As a complex biological system of HBM, exosomes deliver functional cargos of nucleic acids, proteins, and lipids from mothers to breastfeeding infants. The cargos of the exosomes are capable of programming the recipient cells to induce physiological responses in breastfed infants [34]. Although formula milk powder is designed to emulate the composition and function of HBM, it remains incapable of fully substituting for HBM due to the absence of bioactive components and microbiota. Studies have revealed that breastfed and formula-fed infants differ in multiple aspects, including growth patterns, nutritional status, infection frequency, and long-term outcomes such as neurodevelopment, as well as the risks of obesity, diabetes, and cardiovascular disease [35]. Notably, it was reported that exosomes were undetectable in infant formulas, and the exosome-sized vesicles appeared to be casein micelles [36]. In subsequent studies following the removal of casein micelles, researchers revealed that extracellular vesicles (including exosomes) present in infant formulas exhibited structural damage, fragmented backgrounds and reduced levels of extracellular vesicular protein markers, which indicated that these extracellular vesicles were substantially compromised by industrial processing steps, including evaporation, sterilization, and harsh spray drying [37,38]. Although most infant formulas are based on bovine milk, they are largely deficient in milk-derived miRNAs that could potentially influence human development [39]. We therefore hypothesize that HBM exosomes may be a key factor contributing to the differences.

The structure and morphology of exosomes are stable. Exosomes isolated from HBM of 2 months and 6 months after delivery were similar in morphological and phenotypic characteristics. TEM results showed the morphology of HBM exosomes as donut-shaped or cup-shaped, which is consistent with previous research discoveries [16,40,41]. Exosome-specific membrane surface protein expression, such as CD9, CD63 and CD81, suggests similar and phenotypic characteristics.

Zhou et al. [42] identified HBM exosome of Chinese healthy parturients and found half of their top 10 ranked miRNAs were the same as with our results, including miR-148a-3p, miR-30b-5p, miR-146b-5p, let-7a-5p, miR-200a-3p. Six of top 10 miRNAs reported in our study were found among top 20 identified by Liao et al. [43] in HBM from American healthy parturients as follows: miR-148a-3p, miR-30d-5p, miR-30b-5p, let-7g-5p, miR-200a-3p, let-7a-5p. Our results also support that the major miRNA species in HBM are not much affected by maternal ethnicity [22].

Ye et al. [24] presented the lipidomic analysis of HBM-derived extracellular vesicles and identified 11 lipid species, including glycerophospholipids, SM, CER, etc. Jalaludin et al. [44] investigated exosomal lipids from human serum and identified 15 lipid species including SM, CER, TAG, PE, phosphatidylcholine (PC) and so on. Our results were similar with these studies as SM, PE, and PC are components in membranes of exosomes [45].

HBM exosomes exhibit conserved features in terms of their characteristics and composition. Moreover, based on their selectively packaged cargo, exosomes participate in multiple biological processes that support infant growth and development, particularly during the first six months of life.

miRNAs, as one type of epigenetic modification, have the potential to transmit heritable information from mother to the infants [20]. GO enrichment analysis hinted that the predicted target genes of six differentially expressed miRNAs focused on cell junction and participated in signaling regulation through affecting ion binding. The amounts of the various phospholipids in a membrane define the fluidity of the membrane and, consequently, the functions of the embedded proteins [46]. SM as the major phospholipid in HBM may help with low-membrane fluidity, resulting in a stable structure [24]. We found SM (22:0) was the most abundant lipid in group PN-2M and ranked as the top 3 lipid in group PN-6M. Our findings revealed a decrease in PE levels with the extension of the breastfeeding duration, which is consistent with the study by Ye et al. [24] that observed a declining trend in PE concentration from colostrum to mature milk. However, cargo contents of TAG, DAG and FFA were higher in group PN-6M. This may be due to more active cell proliferation at 2 months postpartum and the increasing demand for energy and tissue development at 6 months and afterwards. While the proteins constituting the total milk proteome are largely associated with nutritional pathways and immune functions, the unique proteins of HBM exosomes exhibit greater diversity and are involved in distinct bioactive processes, including cell signaling, cell growth, and cell maintenance [23]. The most interacting differentially expressed protein SEC61A1, engaged on the protein export KEGG enrich pathway, was the main subunit of the Sec61 transporter protein complex. It was responsible for transporting proteins to the endoplasmic reticulum for folding, modification and packaging [47]. Thus, we supposed that the sorting of cargo contents for exosome biogenesis of HBM adapts to the cell and the infant requirements of growth and development.

Infants come through their first critical period from 0 to 6 months after birth for the active proliferation of fat cells. According to WHO child growth standards, the weight of 2-month old boys should be 4.4–7.0 kg, and girls should be 4–6.5 kg, and the weight of 6-month old boys should be 6.4–9.7 kg, and girls should be.5.8–9.2 kg. Gridneva et al. [48] indicated that infant body weight increase within 6 months is mainly due to adipose tissue development. Gale et al. [49] presented a systematic review and meta-analysis of 15 studies and reported the percentage of fat mass and total fat mass were higher in breastfed infants at 3–4 months and 6 months than that of formula-fed infants.

Dominant miRNAs of HBM in our study, seven of top 10, were related to adipose tissue development with different regulation pathways, including miR-148a, miR-21, miR-30d, miR-320, miR146b, miR-30b and let-7 families. MiR-148a modulates adipocyte differentiation of mesenchymal stem cells and inhibits the expression of Wnt1 in the Wnt signaling pathway to improve adipose development [50]. MiR-21 targets the TGF-β signaling pathway, downregulating TGFBR2 expression to enhance adipogenic differentiation [51]. miR-30d promotes adipogenic differentiation of human adipose tissue-derived stem cells and miR-320 stimulates the differentiation of human mesenchymal stem cells into adipocyte lineage by interrupting transcription factor RUNX2 expression [52]. MiR-146b facilitates adipogenesis by inducing post-transcription, specifically inhibiting SIRT1-mediated deacetylating FOXO1 [53]. MiR-30b may positively regulate brown and beige adipogenesis by regulating the receptor-interacting protein 140 [54], while the expression of let-7 family-inhibited clonal expansion and terminal differentiation of pre-adipocytes balances adipogenesis [55]. Such dominant miRNAs of abundance may promote adipogenesis.

Lipidomic analysis revealed a significant upregulation of TAG in group PN-6M, consistent with the TAG level in HBM [56]. Proteomic analysis indicated that differentially expressed proteins were enriched in the biosynthesis of unsaturated fatty acids, fatty acid metabolism and fatty acid elongation processes. In order to support the developmental demands of infant adipose tissue, HBM exosomes may deliver increased levels of TAG and fatty acid metabolism-related proteins to the infant, potentially under the regulation of dominant miRNAs related to adipogenesis. In this manner, key proteins may participate in the regulation of adipogenesis, while TAG may be absorbed along with the larger bulk of fat absorption from milk fat globules and serve as a substrate contributing to adipose tissue development [57].

According to Ages and Stages Questionnaires and growth rules, 2-month-old infants can lift their head independently in the prone position, hold a rod-shaped object and react during play. By 6 months, they can turn over when lying on their back independently, grasp building blocks with both hands and respond to their name. These early developmental changes indicate rapid nervous system maturation. Zhang et al. [58] reported that gray matter volumes of multiple brain regions were significantly higher in breastfed infants than formula-fed infants. Belfort et al. [59] conducted a prospective cohort study and found that breastfed infants had better language development at 3 years of age and higher intelligence at 7 years of age compared with formula-fed infants. Thus, HBM and its exosomes may be associated with both short-term and long-term brain development.

Except adipogenesis regulation-related miRNAs, the other top 10 miRNAs are associated with neurodevelopment. MiR-26a was involved in the regulation of the production of proinflammatory cytokines in microglia, as over-expression of miR-26a may significantly decrease the production of inflammatory cytokines such as tumor necrosis factor α and IL-6 in microglia [60]. Research on PC12 cells demonstrated that over-expression of miR-200a may initiate neuronal differentiation, especially neurite formation, by down-regulating SOX2 and KLF 4 genes [61]. MiR-200a-3p was also implicated in the pathology of Alzheimer’s disease and exerted neuroprotective effects against Aβ-induced toxicity [62]. For the differentially expressed miRNA, miR-214-3p has been reported to ameliorate cognitive dysfunction [63] and protect the neurons, possibly by targeting PTGS2 against perioperative neurocognitive disorders [64]. Low expression of Cav-1 induced by miR-199a-5p contributes to the up-regulated expression of BDNF and VEGF, promoting the neuronal differentiation of NSCs and mediating endogenous neurogenesis in rats after cerebral ischemia [65].

Exosomes have important effects on communicating with neighboring or distant cells, while lipids participate in receptor-mediated endocytosis, macropinocytosis and phagocytosis, promoting the uptake of exosomes [66]. SM, the most abundant lipid in group PN-2M, is a type of fat found in cell membranes and is the major phospholipid in HM, which plays an essential role in myelin integrity and function and axonal maturation and may be implicated in infant brain development [67]. Moreover, about 45% of total phospholipids in the brain are PE [46], which is critical for neurons to survive and function for their role of membrane fusion. The higher expression of PE subclasses in the PN-2M group may be associated with the rapid neurodevelopment that occurs within the first two months of infancy, during which the demand for PE is increased. In general, the function of differentially expressed miRNAs and differentially expressed lipids was consistent with KEGG pathway enrichment analysis of the predicted axon guide and glycerophospholipid metabolism pathway. Differentially expressed proteins were enriched in biosynthesis of unsaturated fatty acids and the fatty acid metabolism pathway to provide lipid materials of the neuron membrane and brain. Thus, HBM exosomes may transport neurodevelopment-related miRNAs and fatty acid metabolism-related proteins to promote the early development of the nervous system and sorting lipids adapting to neural cell requirements.

Correlation network analysis uncovered complex interconnections among a subset of differentially expressed miRNAs, lipids, and proteins. TargetScan and miRDB database searches revealed that neither the SEC61A1 nor the APOB coding genes were among the predicted targets of miR-199a-5p or miR-126-3p. Consequently, the relationships among differentially expressed miRNAs and their associated lipids and proteins in HBM exosomes may not be straightforwardly direct. Further investigations are warranted to elucidate their interrelationships and potential synergistic effects on infant development.

## 5. Strengths and Limitations

This study provides the first comprehensive characterization of temporal variations in the content and composition of exosomes derived from mature milk, spanning the period from 2 to 6 months postpartum. Nevertheless, several limitations of this study should be acknowledged. First, although multi-omics technologies were applied to profile the landscape of HBM exosomes, the exploratory analysis of potential associations with offspring growth and development needs further mechanistic investigation to substantiate these findings. Second, while demographic characteristics were well balanced between the two groups, detailed dietary assessments of lactating mothers were not undertaken. This limitation will be addressed in future studies, and longitudinal follow-up data from the same cohort of mothers would be more beneficial for verifying the variation trend of HBM exosomal miRNA and exploring the underlying mechanisms. Third, our study population was limited to healthy parturients. Future investigations should include parturients with gestational complications, such as gestational diabetes mellitus or preeclampsia, to further clarify the role of HBM exosomes in early life programming.

## 6. Conclusions

It is well acknowledged that the first 1000 days of early life are a crucial period for infant development. HBM, as the initial food of humans, not only provides necessary nutrition, but also transmits genetic information. Our study firstly comprehensively described the multi-omics profile of HBM exosomes 2 months postpartum and 6 months postpartum and revealed the features of HBM exosomes are both conservative and adaptive. The cargoes of HBM exosomes, including miRNAs, lipids, and proteins, may change along with infant development needs, and may especially be associated with adipogenesis and neurodevelopment processes. Uncovering the intrinsic relationships between differentially expressed miRNAs and their corresponding lipids and proteins in HBM exosomes may provide valuable insights for breastfeeding guidance.

## Figures and Tables

**Table 1:** Demographic characteristics of mothers and infants.

	PN-2M (n = 50)	PN-6M (n = 50)	*p* Value *
Mothers			
Delivery age (y)	29.92 ± 3.62	29.36 ± 4.55	0.498
Pre-pregnancy BMI (kg/m^2^)	20.61 ± 2.60	20.31 ± 1.80	0.504
Gestational weight gain (kg)	13.73 ± 5.93	14.12 ± 4.84	0.721
Delivery (natural%)	60.00	58.00	0.375
Smoking (%)	0.00	0.00	—
Alcohol intake (%)	0.00	0.00	—
Infants			
Sex (male%)	54.00%	56.00%	1.000
Birth weight (kg)	3.23 ± 0.56	3.36 ± 0.51	0.207
Birth length (cm)	50.26 ± 2.60	50.22 ± 2.26	0.935
Body weight at sampling (kg)	4.93 ± 1.03	8.10 ± 1.42	<0.001
Body length at sampling (cm)	56.25 ± 4.70	66.77 ± 4.04	<0.001
Head circumference at sampling (cm)	38.37 ± 1.94	42.94 ± 1.92	<0.001

BMI, body mass index; * *p* values were calculated using the 2-sided *t* test for continuous data and the Fisher exact test used for categorical data.

**Table 2:** The positive rate of exosome surface protein expression in HBM exosomes.

Sample No.		Positive Rate of Exosome Membrane Surface Protein Expression (%)			Control
		CD9	CD63	CD81	
PN-2M	641	28.6	16.2	54.8	1.9
	724	57.1	8.4	28.8	0.5
	1810	31.7	16.5	45.1	0.4
	1829	10.0	13.6	15.0	0.2
	1833	19.1	34.1	28.2	1.4
	1877	19.1	19.5	22.3	0.8
	1945	28.2	23.4	33.7	0.6
	1952	24.8	39.3	33.9	0.5
	1964	19.9	11.1	31.8	1.1
	2001	48.6	27.5	62.7	0.2
PN-6M	1511	10.2	5.0	14.9	0.8
	1516	11.7	8.3	26.5	0.4
	1557	16.5	8.0	23.2	0.4
	1567	21.9	15.7	33.6	0.7
	1815	18.0	11.7	32.3	0.4
	1828	19.2	8.5	28.2	0.4
	1860	17.2	11.9	29.6	0.2
	1918	17.1	16.3	21.9	0.3
	1919	28.8	16.3	38.6	0.5
	1978	27.8	14.6	28.2	0.3

**Table 3:** Differential miRNA expression between group PN-2M and PN-6M.

ID	PN-2M Base Mean	PN-6M Base Mean	Log2 Fold Change	*p*
miR-214-3p	3.2619	0.2053	−3.990	0.0087
miR-199a-5p	55.2939	4.9234	−3.489	0.0095
miR-126-3p	22.3336	2.4174	−3.208	0.0129
miR-127-5p	1.0328	0.0101	−6.683	0.0212
miR-144-3p	5.1959	1.9153	−1.440	0.0230
miR-4787-5p	1.3177	0.4231	−1.639	0.0393

**Table 4:** Rank of top 10 lipids in group PN-2M and PN-6M.

RANK	PN-2M	PN-6M
1	SM(22:0)	FFA(16:0)
2	FFA(16:0)	FFA(18:0)
3	FFA(18:0)	SM(22:0)
4	FFA(18:1)	FFA(18:1)
5	FFA(18:2)	FFA(18:2)
6	SM(24:1)	SM(24:1)
7	FFA(14:0)	FFA(14:0)
8	SM(20:0)	TAG(52:0)_FA18:0
9	DAG(16:0/16:0)	SM(20:0)
10	TAG(52:0)_FA18:0	TAG(50:0)_FA18:0

SM, sphingomyelin; FFA, free fatty acid; DAG, diacylglycerol; TAG, tiacylglycerol.

## Data Availability

The data presented in this study are available on request from the corresponding author due to the privacy of participants and novelty of the research.

## Abbreviations

The following abbreviations are used in this manuscript:

HBM	Human breast milk
PBS	Phosphate buffer saline
TEM	Transmission electron microscope
QC	Quality control
AGC	Automatic gain control
IT	Injection time
HCD	High-energy collisional dissociation
NCE	Normalized collision energy
GO	Gene ontology
KEGG	Kyoto Encyclopedia of Genes and Genomes
PCA	Principal component analysis
OPLS-DA	Orthogonal projections to latent structures-discriminate analysis
BMI	Body mass index
FFA	Free fatty acid
DAG	Diacylglycerol
TAG	Triacylglycerol
LPC	Lysophosphatidylcholine
LPE	lysophosphatidylethanolamine
PC	Phosphatidylcholine
PE	Phosphatidylethanolamine
CER	Ceramide
DCER	Dihydroceramide
HCER,	Hexosylceramide
LCER	Lactosylceramide
SM	Sphingomyelin
CE	Cholesteryl esters
PPI	Protein–protein interaction
